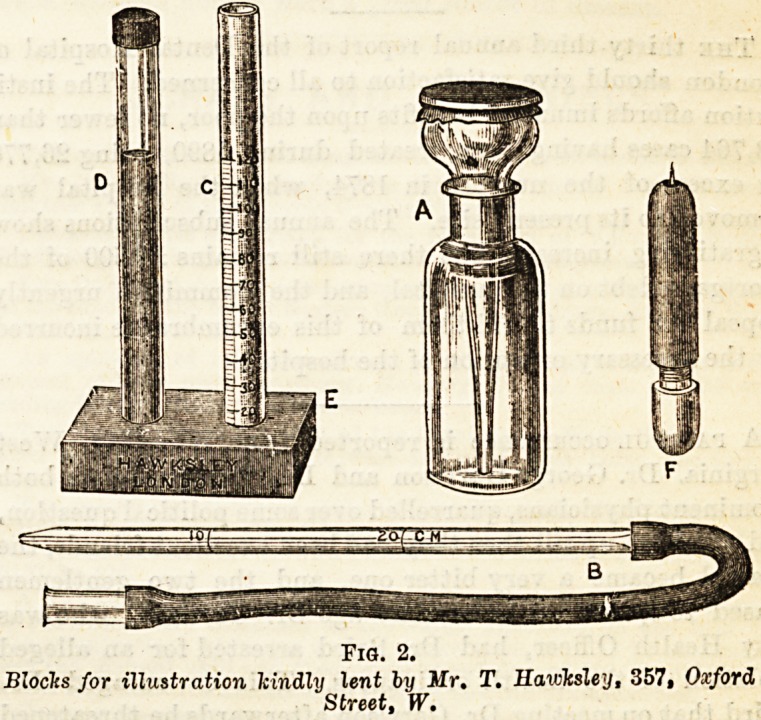# The Microscope in Medicine

**Published:** 1891-03-21

**Authors:** Frank J. Wethered


### The Microscope in Medicine.

By Frank J. Wethered, M.D.

### X.—THE EXAMINATION OF URINARY DEPOSITS.

The ordinary clinical examination of the urine cannot be described here. For the chemical tests for and estimation of albumen, sugar, urea, &c., the reader must consult the text-books, as these subjects hardly come under the head of "The Microscope in Medicine," although they form an important factor in "Clinical Pathology."

Fig. 1.

*Blocks of illustration kindly lent by Mr. T. Hawksley, 357, Oxford Street, W.*

In this article, then, only the examination of urinary deposits will be considered.

In order to collect the deposit, the urine should be allowed to stand for at least twelve hours, protected from dust. The supernatant liquid is then carefully poured off, and portions removed by means of a pipette. This instrument, closed at the upper end by the finger, is passed to the bottom of the glass, and some of the fluid allowed to enter by removing the finger; the pipette is again closed and withdrawn. The superfluous fluid remaining on the tube is wiped off, and the point of the pipette allowed to rest for a few seconds on a glass slide so as to let the heavy particles settle. A drop or two of fluid is then dropped from the tube by slightly moving the controlling finger, and protected by a cover-glass. A great aid is afforded for the recognition of cells, casts, &c., by adding a drop or two of a solution of magenta to the sediment. This stains the solid matter, and thus draws attention to elements which otherwise might escape detection.

For purposes of description it will be best to describe the objects met with in urinary deposits seriatim, rather than to consider the sediments characteristic of various diseases.

The deposits are classified into organised and non-organised. The organised are the corpuscles of blood and pus, the different forms of epithelium from the urinary tract, renal casts, certain parasites, and very rarely portions of new growth. The non-organised sediments are substances which, though normally dissolved in the urine, are thrown out of solution —uric acid, &c. Sometimes abnormal constituents are added, such as leucin and tyrosin. In the present article the non-organised deposits will be described.

Blood.—Red blood corpuscles are found in variable numbers, and always as an abnormal constituent. They may be so few as not to be detected except by the microscope, or may impart to the urine a deep red smoky appearance. They may present their well-known biconcave form and yellowish tinge, but more often they are somewhat altered. If the urine be concentrated they appear shrunken; if it be diluted they lose their colouring matter and look like pale yellow rings.

The blood may come from the urethra, bladder, ureters, or from the pelvis or substance of the kidney. Some information on this point may be derived by the observation of surrounding objects. Thus, if renal casts are also seen, we may conclude that the kidney itself was the cause of the bleeding, but in the absence of these it is extremely difficult to judge by the microscope from which of the parts above named it was derived, and this evidence must be gathered from the grosser features of the case. When intimately mixed with the urine the kidney again is the organ affected. If passed independently of micturition the blood comes from the urethra. When discharged from the bladder it will probably be associated with other signs of cystitis.

Leucocytes (Pus).—A few white blood corpuscles always appear in the urine in health. If there is any doubt as to their nature, this may be ascertained by running under the cover-glass a solution of iodine in iodide of potassium. When occurring in large numbers so as to form pus, the cells present a uniform layer on standing which is easily miscible with the supernatant fluid. They are usually unchanged in form, but sometimes in alkaline urine they swell up and amœboid movements may be observed. They are granular in appearance, but on the addition of acetic acid the granules disappear, and the nucleoli are brought into prominence. They are sometimes seen to contain globules of fat, this especially being the case when an abscess has burst in the urinary tract from other organs.

From a microscopical examination only it is difficult to determine the source of the pus, this being more easily effected by the grosser features and symptoms of the case. But as with blood, some help may be derived from the observance of surrounding objects, especially the various forms of epithelium, which predominate. Thus, if renal epithelium (see below) is present in quantity, it may be assumed that the kidney itself is the source, and in such a case this supposition will probably be corroborated by the appearance of casts composed of pus-cells. The most common cause of pyuria is, of course, cystitis, and then the urine is alkaline and often ammoniacal.

Epithelium.—In the urinary deposit of health, epithelial cells always occur in moderate numbers. This is especially the case in the urine of female patients, where a large number of large polygonal cells, each containing one nucleus, always occur. These are derived from the epithelium of the vagina (Fig. 1, *a.*). They are often grouped together. In the secretion of male patients the cells are much fewer. They are derived from the urethra, and are either polygonal, or oblong, or cylindrical in shape (Fig. 1, *b*), and are much smaller than those of the female. When present in large numbers they denote a catarrhal condition of the parts from which they are derived. The epithelial cells from the bladder ureter and renal pelvis (Fig. 1, *c*) are of varying shape, according to the layers from which they are derived. Those from the superficial layers are mostly polygonal in shape, whilst those from the deeper layers are irregular and somewhat oval. From their form only, no conclusion can be drawn from which of the three parts previously mentioned they come. The cells of the renal epithelium (Fig. 1, *d*), however, are of much more importance. They present every variety of shape, but are chiefly polygonal. They are smaller than those from other parts of the urinary tract, are finely granular, and have relatively larger nuclei. They may cohere one to another so as to form casts of the renal tubules. These will be described more minutely further on. If these cells are present in considerable quantity they denote disease of the kidney, usually nephritis. Occasionally the cells are noticed to be undergoing fatty degeneration, representing a similar process occuring in the kidney.

Perhaps the most important bodies to be sought for in urinary deposits are the casts of the tubules of the kidney, but as these comprise rather an extensive subject, their description will be deferred until the next article. In the present one we will consider very briefly the other organised sediments.

The presence of spermatozoa, each filament consisting of an oval pointed head and long tapering tail, have usually no pathological significance, but are occasionally of great medico-legal importance.

Fragments from the surface of a cancerous ulcer, or portions of villi from a superficial growth of the bladder, may be found occasionally in rare cases.

All urines on standing develop septic micro-organisms, but the only pathogenic microbe of any note is the tubercle bacillus. For their detection the urine is allowed to stand for twenty-four hours, and some of the sediment distributed

Fig. 2.
*Blocks for illustration kindly lent by Mr. T. Hawksley, 357, Oxford Street, W.*

over cover-glasses. On account of the small numbers in which the bacilli usually occur, a great many glasses should be so prepared. After drying each glass is passed three times through the flame of a spirit lamp, and then stained in the same way as described in the examination of sputum. The glasses should be immersed as carefully as possible in the dyes, &c., as the film is very apt to be swept away.

Other parasites occasionally occur, such as the echinococcus (shreds or hooklets), the ova of the Bilharzia hæmatobia, and the Filaria sanguinis hominis (in cases of chyluria). As the space for these articles is necessarily limited, for a description of these parasites the reader is referred to the ordinary text-books.

## Figures and Tables

**Fig. 1. f1:**
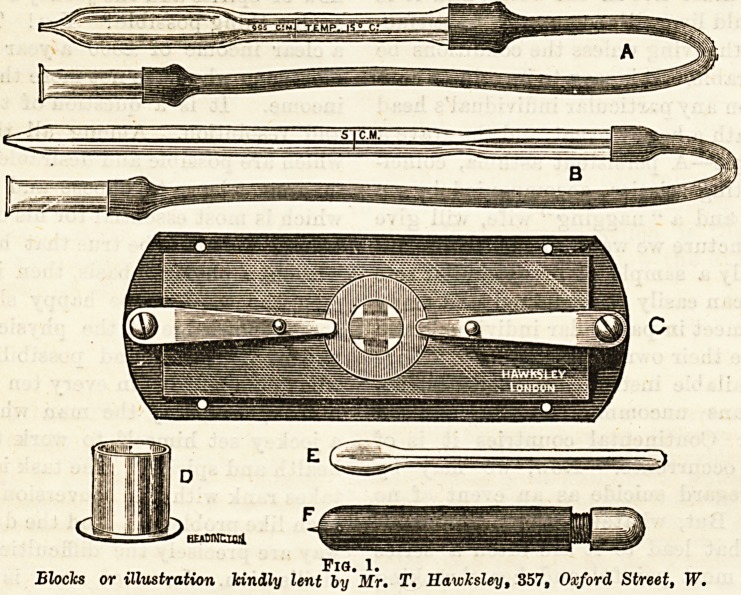


**Fig. 2. f2:**